# Identification of Critical Residues of Linear B Cell Epitope on Goodpasture Autoantigen

**DOI:** 10.1371/journal.pone.0123277

**Published:** 2015-04-13

**Authors:** Xiao-yu Jia, Zhao Cui, Jian-nan Li, Shui-yi Hu, Ming-hui Zhao

**Affiliations:** 1 Renal Division, Department of Medicine, Peking University First Hospital; Institute of Nephrology, Peking University; Key Laboratory of Renal Disease, Ministry of Health of China; Key Laboratory of CKD Prevention and Treatment, Ministry of Education of China, Pekin, PR China; 2 Peking-Tsinghua Center for Life Sciences, Peking, PR China

## Abstract

**Background:**

The autoantigen of anti-glomerular basement membrane (GBM) disease has been identified as the non-collagenous domain 1 of α3 chain of type IV collagen, α3(IV)NC1. Our previous study revealed a peptide on α3(IV)NC1 as a major linear epitope for B cells and potentially nephrogenic, designated as P14 (α3129-150). This peptide has also been proven to be the epitope of auto-reactive T cells in anti-GBM patients. This study was aimed to further characterize the critical motif of P14.

**Methods:**

16 patients with anti-GBM disease and positive anti-P14 antibodies were enrolled. A set of truncated and alanine substituted peptides derived from P14 were synthesized. Circulating antibodies against the peptides were detected by enzyme linked immunosorbent assay (ELISA).

**Results:**

We found that all sera with anti-P14 antibodies reacted with the 13-mer sequence in the C-terminus of P14 (P14c) exclusively. The level of antibodies against P14 was highly correlated with the level of antibodies against P14c (r=0.970, P<0.001). P14c was the core immunogenic region and the amino acid sequence (ISLWKGFSFIMFT) was highly hydrophobic. Each amino acid residue in P14c was sequentially replaced by alanine. Three residues of glycine142, phenylalanine143, and phenylalanine145 were identified crucial for antibody binding based on the remarkable decline (P<0.001) of antibody reaction after each residue replacement.

**Conclusions:**

We defined GFxF (α3142, 143,145) as the critical motif of P14. It may provide some clues for understanding the etiology of anti-GBM disease.

## Introduction

Anti-glomerular basement membrane (GBM) disease is an autoimmune disorder characterized by rapidly progressive glomerulonephritis and in some patients combined with alveolar hemorrhage. The latter is also called Goodpasture’s syndrome [1, 2]. It is a classical autoantibody-mediated disease. The pathogenic role of anti-GBM antibodies was evidenced by passive transfer experiments [3]. The autoantigen of the disease is well-documented as the non-collagenous domain of the α3 chain of type IV collagen [α3(IV)NC1][4, 5], which is also called the Goodpasture autoantigen. Two conformational epitopes have been identified on α3(IV)NC1 as EA (α317–31) and EB (α3127–141) [6]. Further studies identified the critical amino acid residues in EA as Ala18, Ile19, Val27 and Pro28 using recombinant chimeric proteins [7] and the major antibody binding residues in EB as Thr127, Pro131, His134, and Lys141 using phage display technology [8]. These critical residues were clarified on the base of the conformational structures of EA and EB on α3(IV)NC1. However, it remains unknown how these autoantibodies were provoked in the first place.

In recent years, evidence indicating the pathogenic role of T cells in anti-GBM disease has been accumulated [9–12]. In experimental glomerulonephritis models, certain linear nephrogenic T cell epitope shared by B cells was identified and intramolecular epitope spreading was suggested during the process of antibody elicitation [13]. In vivo studies also confirmed that peripheral CD4+ T cells from anti-GBM patients proliferated in response to α3(IV)NC1[14] and the T cell epitopes were further mapped as α369–88 and α3129–148 [15]. In our previous study, we investigated the linear epitopes for B cells in anti-GBM patients using a set of peptides spanning the entire sequence of α3(IV)NC1[16]. P14 (α3127–148) was identified as one of the major linear epitopes recognized by sera from a large cohort of anti-GBM patients. Furthermore, it contained the sequence of EB (α3127–141) and one of the T cell epitopes in anti-GBM patients. These findings impressed P14 as a pivotal epitope on α3(IV)NC1 for eliciting autoimmune response at the early stage of the disease. In fact, we have successfully developed a rat model for anti-GBM disease induced by P14 recently (data unpublished).

In this study, we further characterized the critical residue motif of P14 for B cell recognition. We found that the C-terminus of P14 was the core immunogenic region and three residues were crucial for antibody binding. These results may shed some light on the pathogenesis of anti-GBM disease.

## Materials and Methods

### Sera and patients

Sera from 16 anti-GBM patients with antibodies against P14 were collected from Peking University First Hospital from 1997 to 2008. Sera were obtained on diagnosis and before the start of immunosuppressive therapy or plasmapheresis. All the samples were preserved at -20°C until use. Anti-GBM antibodies were detected in all the 16 samples by enzyme-linked immunoabsorbent assay (ELISA) using purified bovine α(IV)NC1 and recombinant human α3(IV)NC1 as solid phase antigens. Anti-neutrophil cytoplasmic antibodies (ANCA) were screened by indirect immunofluorescence assay and antigen-specific ELISA for antibodies against myeloperoxidase (MPO) and proteinase 3 (PR3) (Euroimmun, Lubeck, Germany). Clinical data at the time of diagnosis as well as during follow-up were collected. Renal pathology data included examinations of light microscopy and direct immunofluorescence microscopy. 24 sera obtained from healthy blood donors were used as normal controls. The research was in compliance of the Declaration of Helsinki and approved by the ethics committee of Peking University First Hospital. Written informed consent was obtained from each participant.

### Preparation of antigens

Peptides were synthesized according to the published sequence of human α3(IV)NC1 as we previously described[16]. P14 (α3 127–148) was divided into three 13-mers overlapping by 8 amino acids, designated as P14a, P14b, and P14c, respectively (Table 1). Each amino acid residue of P14c was then replaced by alanine in a sequential order (Table 2). Peptides were synthesized on an automatic peptide synthesizer using F-moc chemistry (Beijing Scilight Biotechnology Ltd. Co., Beijing, China), and purified by reverse-phase CIS column on a preparative high performance liquid chromatography (HPLC). Purified peptides were analyzed by HPLC for purity and mass spectrometry for the correct sequence. Peptides with purity over 98% were used for further tests.

**Table 1:** Sequences of P14 and P14a-c of α3(IV)NC1.

Peptide	Sequence from N-terminus	Position from N-terminus
P14	TDIPPCPHGWISLWKGFSFIMF	127–148
P14a	TDIPPCPHGWISL	127–139
P14b	CPHGWISLWKGFS	132–144
P14c	ISLWKGFSFIMFT	137–149

**Table 2:** Sequences and antibody response of the peptides with sequential residue substitution by alanine at each position of P14c.

Residue of substitution	Sequence from N-terminus	Position from N-terminus	Mean levels of antibodies(mean ± SD)	P(<0.05)
P14c	ISLWKGFSFIMFT	137–149	0.60±0.26	
I	ASLWKGFSFIMFT	137	0.40±0.20	ns
S	IALWKGFSFIMFT	138	0.43±0.22	ns
L	ISAWKGFSFIMFT	139	0.48±0.22	ns
W	ISLAKGFSFIMFT	140	0.61±0.26	ns
K	ISLWAGFSFIMFT	141	0.52±0.22	ns
**G**	ISLWKAFSFIMFT	**142**	**0.02±0.05**	**<0.001**
**F**	ISLWKGASFIMFT	**143**	**0.15±0.11**	**<0.001**
S	ISLWKGFAFIMFT	144	0.80±0.30	ns
**F**	ISLWKGFSAIMFT	**145**	**-0.03±0.04**	**<0.001**
I	ISLWKGFSFAMFT	146	0.39±0.21	0.021
M	ISLWKGFSFIAFT	147	0.27±0.22	0.006
F	ISLWKGFSFIMAT	148	0.26±0.15	0.015
T	ISLWKGFSFIMFA	149	0.51±0.22	ns

P, levels of antibodies against each substituted peptide were compared with the levels of anti-P14c antibodies, a P value <0.05 was considered significant; ns, no significance

### Detection of linear epitope specificity

Each peptide was diluted at 10μmol/L with 0.05 mol/L bicarbonate buffer (pH 9.6)) and coated onto half of the wells of a polystyrene microtiter plate (Nunc, Roskiled, Denmark). The other half were coated with bicarbonate buffer alone as antigen-free wells to excluded non-specific binding. The volume of each well was 100μL. Incubation was carried out at 4°C overnight. Test sera were diluted at 1:100 in phosphate-buffered saline containing 0.05% Tween-20 and 0.2% BSA (PBST-BSA) and added in duplication to both antigen-coated wells and antigen-free wells at 37°C for 30 min. After washing thrice, alkaline phosphatase-conjugated goat anti-human IgG (Fc specific, Sigma, St Louis, MO, USA) diluted 1:4000 was added. Incubation resumed at 37°C for 30 min. After washing, P-nitrophenyl phosphate (1mg/ml, Sigma) in substrate buffer (1mol/L diethanolamine, 0.5mmol/L MgCl2, PH 9.8) was used as substrate, and color development was measured spectrophotometrically at 405nm (Bio-Rad, Tokyo, Japan) 30 min later. Samples were re-examined when standard errors >10% were found. The net absorbance values were calculated (absorbance values of peptide-coated wells minus the absorbance values of antigen-free wells) for each testing serum on each individual peptide, respectively. 24 sera from healthy blood donors diluted at 1:100 were used to build up the cutoff values using mean + 2 s.d.

### Statistical analysis

Differences of quantitative parameters were assessed using the students’ T test for data that were normally distributed or the nonparametric test for data that were not normally distributed. Pearson’s and Spearman’s rank correlation were performed to analyze the relationship between the levels of antibodies against linear peptides. A *P* value < 0.05 was considered significant.

## Results

### Demographic features and clinical data of patients

16 patients with anti-GBM disease were enrolled in this study. The diagnosis was confirmed by recombinant human α3(IV)NC1 specific ELISA with dual antigen-coated and antigen-free wells. Sera from these patients were detected with positive antibodies against P14 in our previous study [16]. The demographic and clinical data of these patients were shown in Table 3. Of the 16 patients, 8 patients received a kidney biopsy, all of whom were demonstrated with linear IgG deposition along GBM, with or without C3 by direct immunofluorescence. During follow up, 11 (68.8%) patients progressed into end stage renal disease and 6 patients (37.5%) died.

**Table 3:** Demographic data, clinical and pathological parameters of anti-GBM patients with anti-P14 antibodies.

Parameters	% (n)
Age (years)	55.9±19.2
Gender (Male/Female)	9/7
Prodromal infection	68.8% (11)
Hydrocarbon exposure	0% (0)
Smoking	12.5% (2)
Hemoptysis	12.5% (2)
Hemoglobin (g/L)	77.6±18.4
Oliguria/anuria	37.5% (6)
Urinary protein (g/24h)	1.8±1.5
Nephrotic syndrome	6.25% (1)
Gross hematuria	37.5% (6)
Serum creatinine on diagnosis (μmol/L)	563.8±280.5
Positive ANCAaMPO/PR3-ANCA	43.8% (7)6/1
Level of anti-GBM antibodies (U/mL)	50.3±38.8
Crescents in glomeruli (%)	87.3±24.6
Renal survival at one year	37.5% (6)
Patient survival at one year	68.8% (11)

n = 16 patients. ANCA, antineutrophil cytoplasmic antibody; MPO, myeloperoxidase; PR3, proteinase 3; GBM, glomerular basement membrane.

### Identification of the core immunogenic region of P14

**Fig 1:**
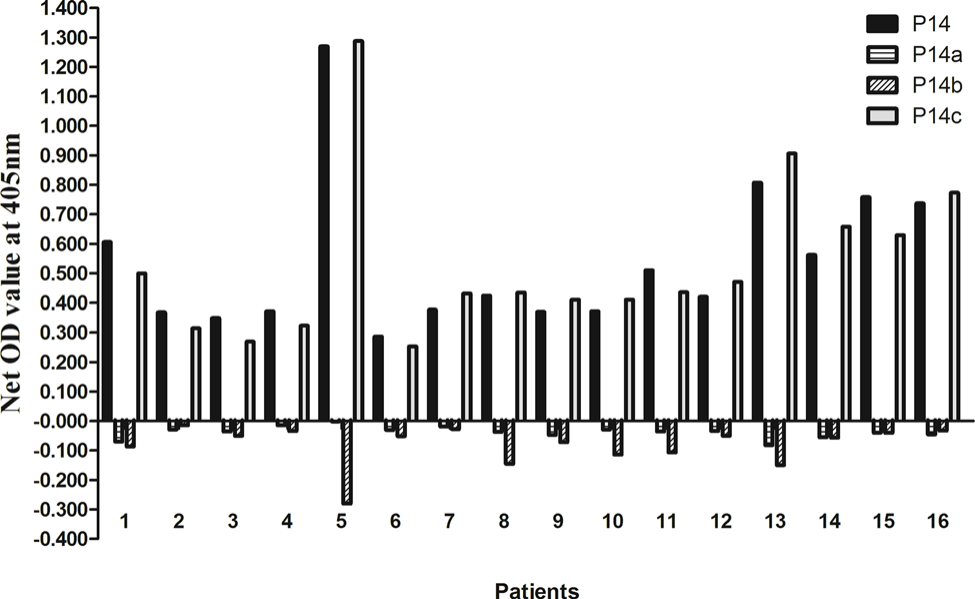
Recognition of P14a, P14b and P14c in anti-GBM patients with anti-P14 antibodies. All sera reacted with P14c exclusively. No serum showed any reactivity to P14a or P14b.

**Fig 2:**
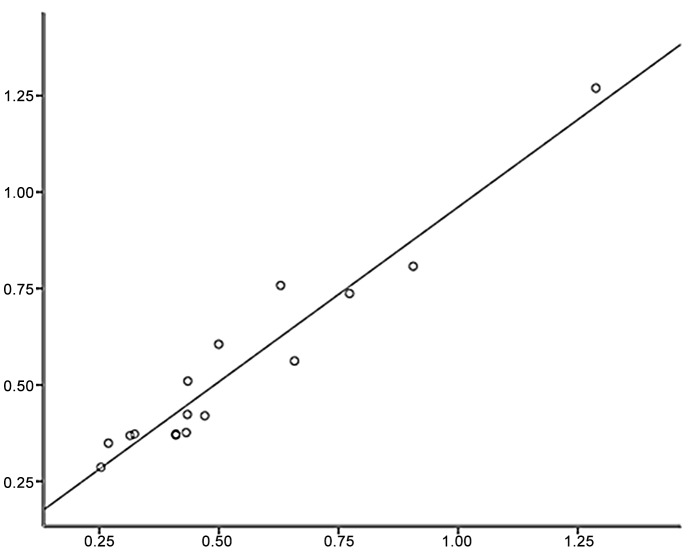
Correlation between the levels of antibodies against P14 and P14c. The level of anti-P14 antibodies was highly correlated with the level of antibodies against P14c.

Three overlapping peptides were synthesized to cover the N-terminus, the central part and C-terminus of P14 (α3127–148), designated as P14a, P14b and P14c, respectively. Each truncated peptide had 13 amino acid residues. Sera from the 16 patients with positive anti-P14 antibodies were detected against the three truncated peptides by ELISA as described above. The results were quite unanimous. All sera reacted with P14c (α3137–149) exclusively. No serum showed any reactivity to P14a or P14b (Fig 1). Furthermore, we found that the level of anti-P14 antibodies was highly correlated with the level of antibodies against P14c as shown in Fig 2 (r = 0.970, P<0.001). Thus, we believed that P14c was the core immunogenic region of P14.

### Mapping critical amino acid residues of P14c

**Fig 3:**
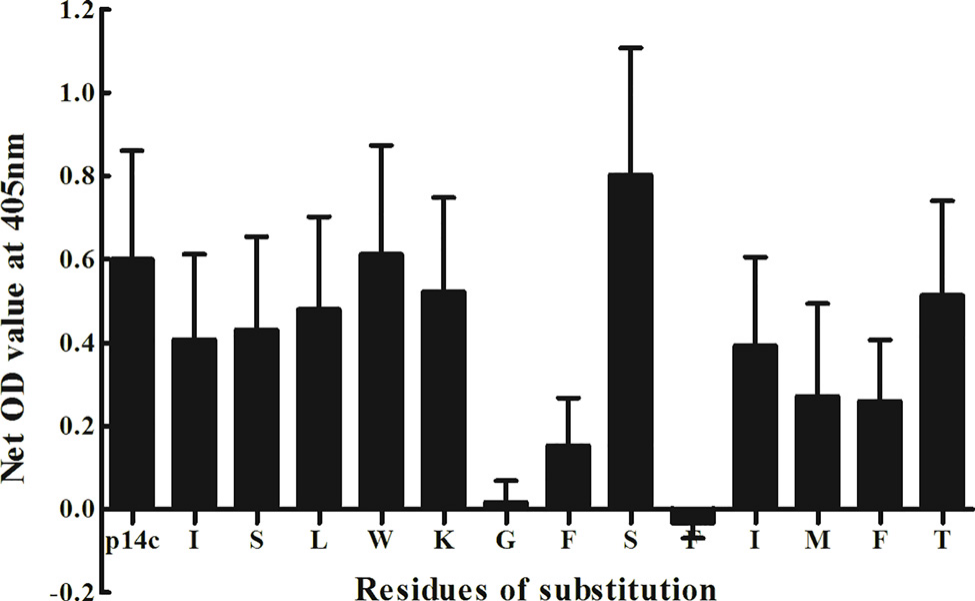
Antibody levels against P14c and the peptides with sequential residue substitution by alanine at each position of P14c. Sera of the 16 anti-GBM patients were detected against each substituted peptides by ELISA. The substitutions at positions 142, 143 and 145 of P14c had significant affect on the antigenicity of P14c (P<0.001).

The critical residues of P14c (α3137–149) were mapped using a set of alanine-substituted peptides. Each amino acid residue in P14c was sequentially replaced with alanine. A total of 13 substituted peptides were synthesized accordingly. Sera of the 16 patients were detected against each substituted peptide. We found that the substitutions at positions 142, 143 and 145 had the most significant affect on the antigenicity of P14c (Table 2). The three corresponding residues were glycine142, phenylalanine143, and phenylalanine145. After each replacement of these residues, the levels of antibody reaction declined remarkably (Fig 3). Thus GFxF (α3142, 143, 145) were defined as the critical residue motif for P14c antigenicity. Besides, the substitutions of residues isoleucine 146, methionin 147, phenylalanine 148 also caused reduction of antibody response.

## Discussion

In our previous study, P14 (α3127–148) was identified as a major linear epitope for B cells in a large cohort of anti-GBM patients [16]. Since it was also a formerly defined T cell epitope [15], we speculated that P14 might initiate the disease by activating both humoral and cellular responses, provoke epitope spreading, and finally induce kidney injury [16]. We have successfully developed a rat model for anti-GBM disease induced by P14 recently (data unpublished). In the present study, we further defined the critical residue motif of P14 for B cell recognition. The characterization of the potential nephrogenic epitope on Goodpasture autoantigen is of vital importance for understanding the etiology of the disease. It might also enable us to investigate the possibility of molecular mimicry as a mechanism of self-tolerance disruption in human anti-GBM disease [17].

We found that the C-terminus of P14 was the core immunogenic region with 13 amino acid residues. The role of each amino acid residue in this region was then determined using a set of peptides with sequential amino acid substitution. It was shown that the antigenicity of P14c was completely lost by the substitution of glycine142 or phenylalanine145 to alanine, and remarkably decreased by the substitution of phenylalanine143 to alanine. Thus GFxF (α3142, 143, 145) was defined as the critical residue motif for P14c antigenicity. Besides, other residues in P14c might modulate the binding capacity of antibodies to a lesser extent. For example, the substitutions of isoleucine 146, methionin 147, or phenylalanine 148 also reduced the antibody response. However, antigenicity of P14c was still detectable after these substitutions, which indicates a participation role of these residues for P14c antigenicity but not an essential role (critical residues).

Interestingly, P14c proved difficult to be synthesized because of its hydrophobicity. Furthermore, two of the three critical residues of P14c (phenylalanine143 and 145) were also hydrophobic. It suggested that the immunogenic region of the epitope had a propensity to be buried within α3(IV)NC1 and normally inaccessible for antibody binding. Furthermore, the C-terminus of P14 was inclined to be cleaved substantially by proteases in early antigen processing, which prevented its constitutive presentation at sufficient levels to T cells [18]. The cryptic nature of the epitope confers the risk of self-tolerance disruption on it when autoimmune response initiate at the onset of the disease. Besides, P14c had a high binding affinity of MHC-DRB1*1501, which is a susceptible genetic marker of anti-GBM disease for Caucasian[19], Japanese[20] and Chinese [21]. Therefore, we believed that the C-terminus of P14 is the nephrogenic region on Goodpasture autoantigen.

Intramolecular epitope spreading of B cells has been demonstrated in experimental anti-GBM nephritis models when antibody response spread to epitopes on α3(IV)NC1 external to the initial one. EB (α3127–141) is one of the two conformational epitope on α3(IV)NC1, which was proved to be of significant clinical importance[22]. The amino acid sequence of P14c and EB was partially overlapped. Thus, we proposed that from P14c the intra-molecular epitope spreading might start and antibodies against conformational epitope EB was initiated.

Recently, Ooi et al identified a nephrogenic T cell epitope on murine α3(IV)NC1 as α3136–146 using HLA-DRB1*1501 transgenic mice [23], which was similar to the sequence of P14 in our study. They mapped the critical residues of this epitope to valine138, tryptophan141, glycine143, and phenylalanine144. Two of the three critical residues of P14 defined in the present study were the same as that of the murine T cell epitope. Considering the high conservation of α3(IV)NC1 between mouse and human, we speculated that the critical residues of P14 for T cell recognition might be the corresponding amino acids as isoleucine137, tryptophan140, glycine142 and phenylalanine143 (human numbering). Therefore, the last two critical residues of P14 might be crucial for both B cell and T cell recognition in human anti-GBM disease.

It is reasonable to find that the critical residues for T cell and B cell response are similar, or overlapped. This phenomenon was also described in WKY rats by Wu J et al[24], who defined the sequence of SQTTANPSCPEGT as the mutual immunogenic region for both T cells and B cells [25]. This could be explained by the following reasons. Firstly, during the process of autoimmunity, there are complicated interactions between T cells and B cells [26–29]. Antigen presented by B cells controls the functional response of antigen-specific T cells [30, 31]. It has been reported in autoimmune thyroiditis that autoantibodies against thyroid peroxidase (TPO) modulate the T cell epitope repertoire [32]. Apart from professional antigen-presenting cells, such as dendritic cells, B cells could present antigens more avidly with BCRs for the recognition of T cells [33]. Therefore, the pathogenic epitope of an autoantigen for both T and B cells might be the same, or at least adjacent. Secondly, according to the published crystal structures of MHC-peptide-TCR complex, the anchor motif of HLA-DRB1*1501 consists of a nonaromatic, hydrophobic anchor (L, V, or I) at position 1 and a bulky hydrophobic residue (F or Y) at position 4[34]. It partially overlaps the critical motif of P14 (GFxF) for B cell recognition in the present study. However, the degeneracy of TCR permits widely TCR binding-cross reactivity[34, 35], which also supports the diversity of the critical motif for T cell and B cell recognition.

In conclusion, the C-terminus of P14, or P14c, was the core immunogenic region and highly hydrophobic. The critical residues of P14c were mapped as glycine142, phenylalanine143, and phenylalanine145. The critical residues for T and B cell recognition were partially overlapped, which may provide some clues in further investigation of the pathogenesis of anti-GBM disease.
